# Simple extraction methods that prevent the artifactual conversion of chlorophyll to chlorophyllide during pigment isolation from leaf samples

**DOI:** 10.1186/1746-4811-9-19

**Published:** 2013-06-19

**Authors:** Xueyun Hu, Ayumi Tanaka, Ryouichi Tanaka

**Affiliations:** 1 Institute of Low Temperature Science, Hokkaido University, Sapporo, 060-0819, Japan; 2 CREST/JST, Hokkaido University, Sapporo, 060-0819, Japan

**Keywords:** Chlorophyll extraction, Chlorophyllide, Chlorophyllase

## Abstract

**Background:**

When conducting plant research, the measurement of photosynthetic pigments can provide basic information on the physiological status of a plant. High-pressure liquid chromatography (HPLC) is becoming widely used for this purpose because it provides an accurate determination of a variety of photosynthetic pigments simultaneously. This technique has a drawback compared with conventional spectroscopic techniques, however, in that it is more prone to structural modification of pigments during extraction, thus potentially generating erroneous results. During pigment extraction procedures with acetone or alcohol, the phytol side chain of chlorophyll is sometimes removed, forming chlorophyllide, which affects chlorophyll measurement using HPLC.

**Results:**

We evaluated the artifactual chlorophyllide production during chlorophyll extraction by comparing different extraction methods with wild-type and mutant Arabidopsis leaves that lack the major isoform of chlorophyllase. Several extraction methods were compared to provide alternatives to researchers who utilize HPLC for the analysis of chlorophyll levels. As a result, the following three methods are recommended. In the first method, leaves are briefly boiled prior to extraction. In the second method, grinding and homogenization of leaves are performed at sub-zero temperatures. In the third method, N, N’-dimethylformamide (DMF) is used for the extraction of pigments. When compared, the first two methods eliminated almost all chlorophyllide-forming activity in *Arabidopsis thaliana, Glebionis coronaria, Pisum sativum* L. and *Prunus sargentii* Rehd. However, DMF effectively suppressed the activity of chlorophyllase only in Arabidopsis leaves.

**Conclusion:**

Chlorophyllide production in leaf extracts is predominantly an artifact. All three methods evaluated in this study reduce the artifactual production of chlorophyllide and are thus suitable for pigment extraction for HPLC analysis. The boiling method would be a practical choice when leaves are not too thick. However, it may convert a small fraction of chlorophyll *a* into pheophytin *a*. Although extraction at sub-zero temperatures is suitable for all plant species examined in this study, this method might be complicated for a large number of samples and it requires liquid nitrogen and equipment for leaf grinding. Using DMF as an extractant is simple and suitable with Arabidopsis samples. However, this solvent cannot completely block the formation of chlorophyllide in thicker leaves.

## Background

Chlorophyll analysis has been conducted in numerous studies due to the importance of this pigment in the physiology of plants. Chlorophyll is involved in the absorption and transfer of light energy, and electron transfer, all of which are vital processes in photosynthesis. Chlorophyll content can change in response to biotic and abiotic stresses such as pathogen infection [1], and light stress [2,3]. Thus, quantification of chlorophyll provides important information about the effects of environments on plant growth [4-8].

Historically, spectroscopic methods have been most frequently used for chlorophyll measurement because they provide a quick, accurate and inexpensive estimation of chlorophyll concentration [9-11]. However, conventional spectroscopic methods, where bulk photosynthetic pigments are measured in the same cuvette, have limitations in their ability to simultaneously measure multiple photosynthetic pigments due to the overlapping absorption spectra of these pigments. For this reason, it has become more common to separate photosynthetic pigments by high-pressure liquid chromatography (HPLC) prior to spectrophotometric analysis [12,13]. When separating pigments by HPLC, extra care must be taken since HPLC analysis is prone to the artifactual modification of pigments. In particular, cleavage of the phytol chain of chlorophyll molecules readily occurs with the use of common extraction solvents such as 80% acetone [14]. The products of chlorophyll hydrolysis are chlorophyllide and free phytol*.* Since chlorophyllide has the same absorption spectra as chlorophyll in the visible light spectrum and phytol does not, cleavage of the phytol chain does not affect the values obtained using conventional spectroscopic methods of chlorophyll determination when samples are extracted with organic solvents. However, due to the polar nature of chlorophyllide, it is readily separated from chlorophyll with HPLC, and thus the artifactual formation of chlorophyllide can result in erroneous data using HPLC-based determination of chlorophyll concentration.

Conversion of chlorophyll to chlorophyllide induced by the extraction agent reduces the apparent concentration of chlorophyll in samples. It is usually difficult to distinguish whether or not the chlorophyllide detected during HPLC analysis is an artifact or a natural product. In fact, chlorophyllide has been considered a natural product in leaves without examining the basis of its formation [15,16]. In order to avoid possible misinterpretation of chlorophyll levels, it is essential to employ extraction methods that result in a minimal amount of conversion of chlorophyll to chlorophyllide.

**Figure 1:**
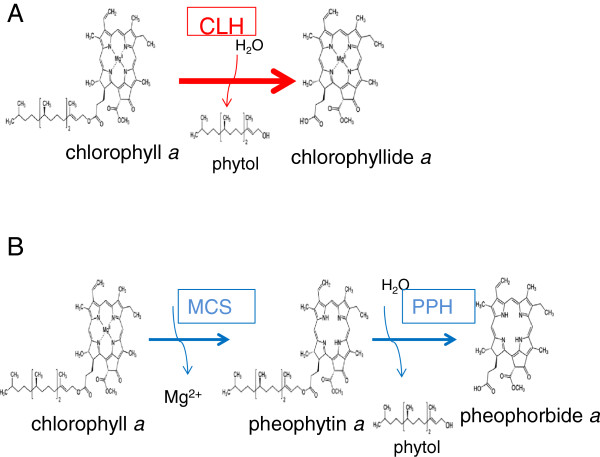
**Comparison of the CLH reaction and a proposed *in vivo *degradation pathway of chlorophyll *a. *A**. CLH catalyzes the hydrolysis of the ester bond of chlorophyll to form chlorophyllide and phytol. **B**. An *in vivo* degradation pathway of chlorophyll *a* proposed by Hörtensteiner and coworkers [29]. MCS denotes magnesium dechelating substances. PPH denotes pheophytinase.

It has been reported that the hydrolase enzyme, chlorophyllase (CLH) catalyzes the formation of chlorophyllide during pigment extraction [17] (Figure 1A). This enzyme is unusually stable in high concentrations of organic solvents such as 50-70% aqueous acetone [18,19]. Higher plants contain one or two isoforms of this enzyme [20] and Arabidopsis has two CLH isoforms encoded by *CLH1* and *CLH2* genes, respectively [21]. *CLH1* encodes the isoform of CLH that accounts for the majority of CLH in Arabidopsis leaves. *CLH1* gene expression is significantly upregulated by methyl-jasmonate (MeJA), a phytohormone mediating various biotic and abiotic signaling pathways [22]. In contrast, *CLH2* is constitutively expressed and only represents a minor fraction of CLH activity [23]. In the present study, we assessed how much chlorophyllide is formed during pigment extraction compared to the amount that naturally occurs in leaves. In a subsequent analysis, we then examined whether or not CLH is involved in chlorophyllide formation during extraction by comparing its formation in leaves of wild-type and an Arabidopsis mutant which is deficient in CLH activity. Collectively, these experiments indicated that the majority of chlorophyllide detected in extracts obtained using 80% acetone or pure acetone is produced during pigment extraction through the reaction catalyzed by CLH. We also compared three different methods of pigment extraction that were previously reported in literature. Bacon and Holden [17] reported that CLH activity could be suppressed by boiling leaves for a period of 5 min. They also indicated, however, that the boiling treatment also removes Mg^2+^ from chlorophyll [17]. We found that, in the case of Arabidopsis leaves, CLH can be inactivated and Mg^2+^ removal from chlorophyll can be reduced when samples were boiled for only 5 sec. In the method of Schenk et al. [23], leaves were first ground into powder in liquid nitrogen and pigments were subsequently extracted in buffered acetone cooled to −20°C. We found this method is very efficient when processing a relatively small number of samples. Finally, we tested the use of N, N’-dimethylformamide (DMF) as an extraction agent to eliminate the formation of chlorophyllide during sample preparation. Although Moran and Porath [24] reported that chlorophyll is stable in this solvent, they did not characterize the effect of DMF on chlorophyllide formation. In our study, DMF was capable of extracting pigments without enabling the conversion of chlorophyll to chlorophyllide in Arabidopsis, however, for the other species which we have tested in this study, DMF cannot completely suppress the activity of CLH. Collectively, all three methods (boiling leaf sample, freezing leaf samples in liquid nitrogen with the use of pre-cooled acetone, and the use of DMF as an extraction agent) were superior to the methods only using 80% or pure acetone for the extraction of photosynthetic pigments. It is important to understand advantages and disadvantages of each method and choose an appropriate one for each plant species and for the purpose of pigment analysis.

## Results

### Use of 80% and pure acetone as extraction solvents results in the formation of chlorophyllide

**Figure 2:**
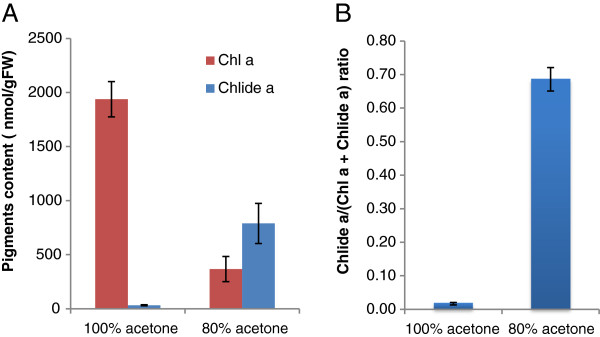
**Formation of chlorophyllide *a *by the extraction of chlorophyll with pure or 80% buffered acetone.** Frozen leaves were immersed in pure acetone or 80% acetone containing 20% (v/v) Tris–HCl, pH 8 and then incubated in these solvents for 12 h at 4°C in the dark. Pigments were subsequently extracted by grinding with stainless steel beads as described in the Methods section. **A**. Levels of chlorophyll *a* and chlorophyllide *a* per gram fresh weight of leaves. **B**. Chlorophyllide *a* levels in sample extracts expressed as the ratio of cholorophyllide *a* to the sum of chlorophyll *a* and chlorophyllide *a.* Chl *a*, chlorophyll *a*. Chlide *a*, chlorophyllide *a*. Error bars indicate standard deviations. Sample size, n = 3.

It was our objective to provide a simple and reliable method to extract chlorophyll for HPLC analysis that would be free from artifacts. In order to achieve this goal, we started with one of the simplest methods to extract pigments and attempted to improve it. We first compared two conventional methods in which pigments are extracted from Arabidopsis leaf samples by soaking them in 80% or pure acetone for 12 hours at 4°C. Since CLH has been reported to be active in aqueous acetone but precipitated in pure acetone [18,19], it was expected that chlorophyllide would only be produced in the 80%-acetone extracts. In line with this expectation, we determined that nearly 70% of the combined chlorophyll and chlorophyllide *a* content was composed of chlorophyllide *a* in the 80%-acetone extracts (Figure 2). In contrast, only a small amount of chlorophyllide *a* was produced in pure acetone (Figure 2). Therefore, it is likely that most of the chlorophyllide *a* detected in 80% acetone was formed during extraction or after extraction. Since chlorophyll *a* is much more abundant than chlorophyll *b* and the trend of chlorophyllide *b* formation was similar to that of chlorophyllide *a*, we only describe the results on chlorophyll *a* and chlorophyllide *a* in the present study.

**Figure 3:**
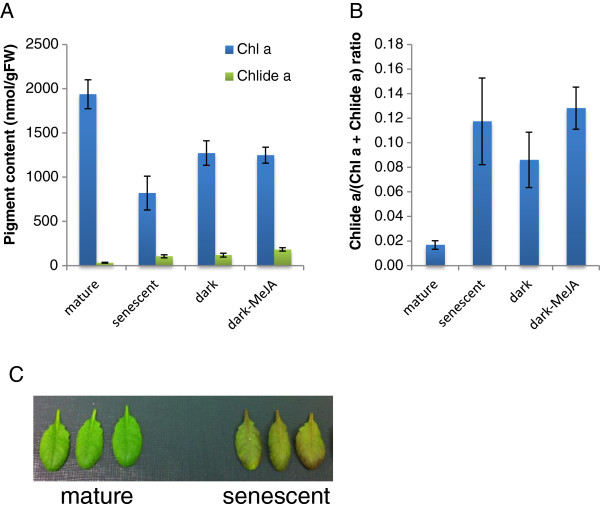
**Chlorophyllide formation during pigment extraction from senescent or MeJA-treated leaves.** Pigments extracted from mature leaves (7th to 9th leaves counting from the bottom of the plant) of wild-type (WT) Arabidopsis plants under four different conditions: leaves collected from 4-week-old plants (“mature”), leaves from 8-week-old plants (“senescent”), leaves from 4-week-old plants where the removed leaves were incubated in complete darkness on filter paper saturated with 3 mM MES buffer (“dark”) or the same buffer plus 50 μM MeJA (“dark-MeJA”). Pigments were extracted by immersing the leaves in pure acetone that was cooled to 4°C and the extracts were subsequently analyzed by HPLC (see the Methods section for detail). **A**. Levels of chlorophyll *a* and chlorophyllide *a* per gram fresh weight of leaves. **B**. Chlorophyllide *a* levels in sample extracts expressed as the ratio of chlorophyllide *a* to the sum of chlorophyll *a* and chlorophyllide *a.* Chl *a*, chlorophyll *a*. Chlide *a*, chlorophyllide *a*. Error bars indicate standard deviations. Sample size, n = 3. **C**. The photograph below the bar graphs illustrates the 4-week-old (left) and 8-week-old (right) leaves used in these analyses.

CLH activity increases in leaves that are either senescent [25] or wounded [16], and in response to MeJA treatment [21]. Thus, we examined chlorophyllide formation using naturally-senescent leaves from 8-week-old Arabidopsis plants, those in which senescence was induced by a 4-day dark treatment, and those in which chlorophyll breakdown was induced by MeJA. Eight to ten percent of the chlorophyll content in naturally-senescent, dark-treated, and MeJA-treated leaves was composed of chlorophyllide (Figure 3) even when pure acetone was used. These results indicate that pure acetone does not sufficiently suppress the formation of chlorophyllide during chlorophyll extraction.

### CLH is responsible for the formation of chlorophyllide during chlorophyll extraction

**Figure 4:**
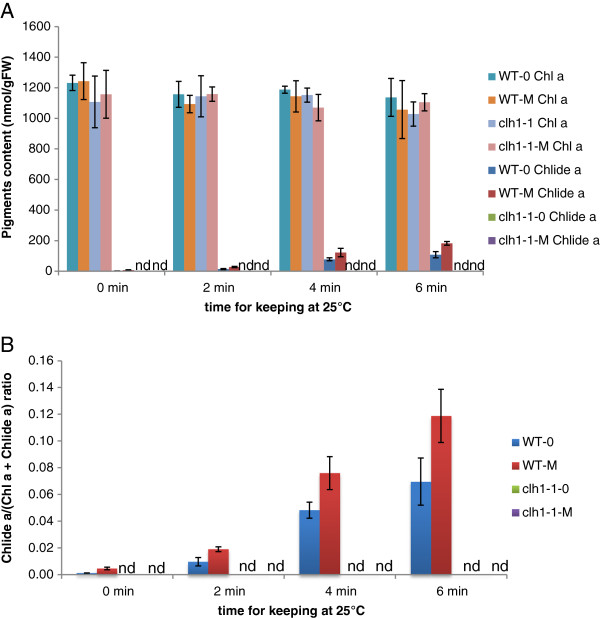
**Time course of chlorophyllide *a *formation during pigment extraction.** Chlorophyll was extracted by immersing leaves in pure acetone as described in the “Time-course experiments” subsection of the Methods section. **A**. Levels of chlorophyll *a* and chlorophyllide *a* per gram fresh weight of leaves. **B**. Chlorophyllide *a* levels in sample extracts expressed as the ratio of chlorophyllide *a* to the sum of chlorophyll *a* and chlorophyllide *a.* Chl *a*, chlorophyll *a*. Chlide *a*, chlorophyllide *a*. Error bars indicate standard deviations. Sample size, n = 3. n.d. = not detected.

The experiments described above indicated that the majority of chlorophyllide is formed during extraction. In the next series of experiments, we examined the time-course of chlorophyllide formation during acetone extraction. We also tested whether or not CLH was involved in chlorophyllide formation in pure acetone by comparing chlorophyllide formation during extraction using Arabidopsis leaves from wild type (WT) and a *clh1-1* mutant that lacks the major isoform of CLH. After immersing leaves in pure acetone, leaves were incubated in pure acetone for up to 6 min (Figure 4). In this series of experiments, chlorophyllide formation was also compared in leaves from WT plants that had been either treated or not with 50 μM MeJA because the CLH activity is more evident after MeJA treatment (Figure 4).

Production of chlorophyllide was negligible unless incubation was at an ambient temperature (Figure 4). Increasing incubation time at an ambient temperature resulted in elevated amounts of chlorophyllide (Figure 4). MeJA-treated WT leaves yielded a higher amount of chlorophyllide (up to 12% of the total chlorophyll plus chlorophyllide after 6 min of incubation) compared to non-treated WT leaves. In contrast, extracts from leaves from *clh1-1* plants only contained a trace amount of chlorophyllide with or without MeJA treatment (Figure 4). These results are consistent with the report of Schenk et al. [23], in which they detected a much lower level of chlorophyllide in acetone extracts of dark-incubated *clh1-1* leaves compared to extracts from dark-incubated wild-type leaves. These results indicate that the major isoform of CLH is responsible for chlorophyllide formation during extraction.

### Stability of chlorophyll and chlorophyllide in pure acetone

In our next line of investigation, we tested the stability of chlorophyll and chlorophyllide in pure acetone. Chlorophyllide production was evident from an extraction of WT leaves in pure acetone at ambient temperatures for 6 min (Figure 4 and Table 1). Specifically, the pigments were extracted by grinding leaves in pure acetone with stainless steel balls and the extracts were separated from cell debris by centrifugation. This procedure yielded approximately 200 nmol/gFW (fresh weight) chlorophyllide in the extract. In contrast, chlorophyllide levels were less than 10 nmol/gFW in the absence of the 2 – 6 min incubation. Extracts were also maintained for one day in darkness at room temperature and their chlorophyll and chlorophyllide levels were measured using HPLC. No significant changes in chlorophyll or chlorophyllide levels were observed in the one-day-old extracts (Table 1). Therefore, it is likely that the majority of chlorophyllide detected in the extracts were formed during the extraction or homogenization procedures.

**Table 1:** **Stability of chlorophyll *a *and chlorophyllide *a *after extraction with pure acetone**

	**0 day**		**1 day**	
**Time**	**Chlide *a***	**Chl *a***	**Chlide *a***	**Chl *a***
**(min)**	**(nmol/gFW)**	**(nmol/gFW)**	**(nmol/gFW)**	**(nmol/gFW)**
0	9	1284	9	1317
	5	1107	7	1167
	8	1338	6	1340
6	211	979	203	983
	191	1249	203	1329
	168	1052	171	1081

Mature Arabidopsis leaves (7th - 9th leaves counting from the bottom of the 4-week-old plants) were treated with 50 μM MeJA and were kept in complete darkness for 3 days. Chlorophyll was extracted by immersing leaves in pure acetone as it is described in the “Time-course experiments” subsection of the Methods section. “Time” indicates the incubation time at room temperature. After removing the residual leaf tissue by centrifugation, the extracts were incubated for 24 hours at room temperature. Each line represents a single experiment with an extract from a single leaf, and experiments were repeated three times in the same conditions. Values represent chlorophyllide *a* or chlorophyll *a* concentrations per gram fresh weight of leaves. Chl *a*, chlorophyll *a*. Chlide *a*, chlorophyllide *a*.

### Chlorophyllide formation is suppressed with rapid boiling of leaves

In order to provide a simple chlorophyll extraction method that is less affected by CLH activity, we assessed if CLH activity could be inactivated by boiling leaves. Bacon and Holden [17] have already reported that CLH activity can be suppressed by boiling leaves for 5 min. However, they also found that this treatment destroys some pigments. Therefore, we examined whether shorter (approximately 5 or 10 sec) periods of boiling can adequately suppress CLH activity while avoiding pigment decomposition. In this experiment, mature Arabidopsis leaves were sprayed with 50 μM MeJA and subsequently harvested. After collection they were dipped in boiling water for 5 or 10 sec, and then soaked in pure acetone. For pigment extraction, leaves were homogenized in acetone using stainless beads or kept in pure acetone overnight at 4°C.

**Figure 5:**
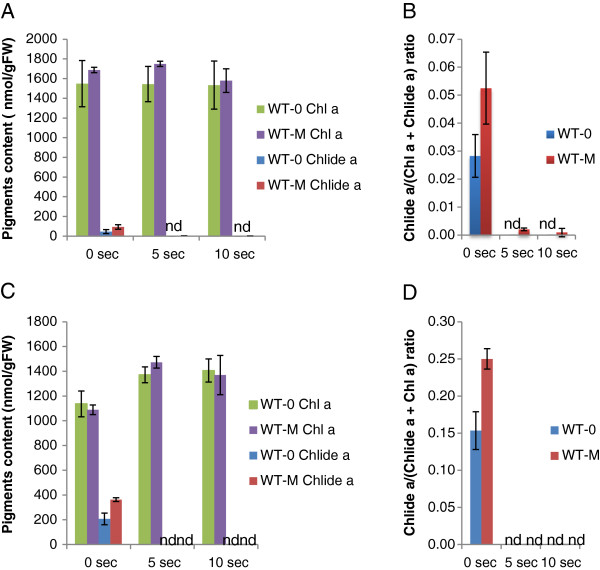
**Effect of quick boiling on the formation of chlorophyllide *a *during extraction.** Mature leaves (7th to 9th leaves counting from the bottom of the plant) were collected from 4-week-old wild-type plants that had been sprayed with 100 μM MeJA and kept for 3 days at the same growth conditions. The collected leaves were immersed in pure acetone at room temperature directly or were boiled for 5 and 10 sec respectively before they were immersed in pure acetone, then they were ground with stainless steel beads by vigorous shaking (**A** and **B**). Alternatively, pigments were extracted by immersing leaves in pure acetone at 4°C for 12 hours (**C** and **D**). **A** and **C**, Levels of chlorophyll *a* and chlorophyllide *a* per gram fresh weight of leaves. **B** and **D**, Chlorophyllide *a* levels in sample extracts expressed as the ratio of chlorophyllide *a* to the sum of chlorophyll *a* and chlorophyllide *a.* Chl *a*, chlorophyll *a*. Chlide *a*, chlorophyllide *a*. Error bars indicate standard deviations. Sample size, n = 3.

**Figure 6:**
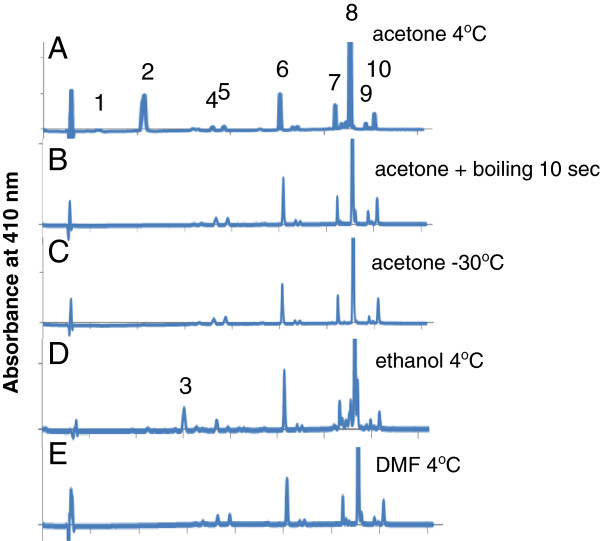
**The elution profiles of pigments from extracts separated by HPLC.** Representative HPLC profiles from each experiment are shown. **A**. HPLC profile of pigments extracted by immersing wild-type (WT) leaves in pure acetone for 12 hours at 4°C. **B**. An HPLC profile of WT pigments extracted by the quick boiling method. **C**. HPLC profile of pigments extracted from leaves of WT plants by immersing leaves in pure acetone for 96 hours at −30°C. **D**. HPLC profile of pigments extracted from leaves of WT plants by immersing leaves in ethanol at 4°C for 12 hours. **E**. HPLC profile of pigments extracted from leaves of WT plants by immersing leaves in DMF at 4°C for 12 hours. Peak 1, chlorophyllide *b.* Peak 2, chlorophyllide *a,* Peak 3, pheophorbide *a.* Peak 4, neoxanthin. Peak 5, violaxanthin*.* Peak 6, lutein. Peak 7, chlorophyll *b.* Peak 8, chlorophyll *a.* Peak 9, pheophytin a. Peak 10, β-carotene.

Leaves from WT plants that were homogenized without boiling yielded 94 nmol/gFW and 46 nmol/gFW chlorophyllide *a* when they were treated or not with MeJA, respectively, prior to extraction (Figure 5A). In contrast, chlorophyllide *a* was below a detectable level in leaves that were not treated with MeJA when the leaves were boiled for 5 or 10 sec before chlorophyll extraction. When MeJA-treated WT leaves were boiled for 5 or 10 sec prior to extraction, only 3.6 and 1.4 nmol/gFW chlorophyllide *a* were detected, respectively (Figure 5A). The combined sum of chlorophyll and chlorophyllide were not affected by boiling (Figure 5A and B), indicating that the significant pigment losses, observed when leaves were subjected to 5 min boiling [17], did not occur when the brief boiling procedure was used. The overall profiles of detectable photosynthetic pigments obtained by HPLC were not altered by boiling except for pheophytin *a.* This pigment increased slightly from 30 nmol/gFW to 50 nmol/gFW in boiled samples. These levels represented approximately 0.2% to 0.3% of total chlorophyll *a* levels, and are almost negligible in the HPLC profiles (Figure 6B).

To further simplify the extraction method, boiled leaves were kept overnight in pure acetone at 4°C. Chlorophyllide formation in both MeJA-treated and non-treated boiled WT leaves were negligible after overnight incubation (Figure 5C and 5D), indicating that rapid boiling almost completely inactivated CLH activity.

### Pure acetone extraction at sub-zero temperatures

**Figure 7:**
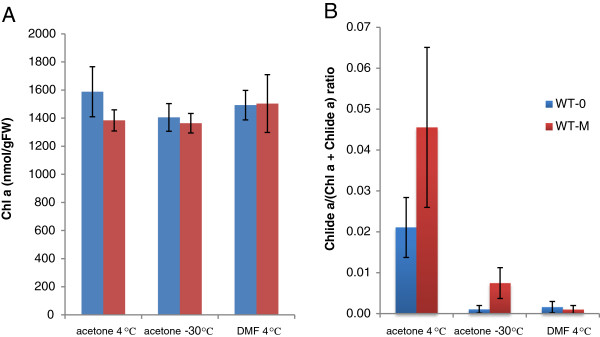
**Pigment extraction at −30°C in pure acetone and at 4°C in DMF.** Mature Arabidopsis leaves (7th to the 9th leaves counting from the bottom of the plant) that were treated with or without MeJA (see the Method section for the detail) were harvested and their pigments were extracted by three different methods. In the first two methods, leaves were immersed in pure acetone at 4°C for 12 hours (“acetone 4°C”) and at −30°C for 96 hours (“acetone −30°C”) respectively. In the third method, leaves were immersed in DMF at 4°C for 12 hours (“DMF 4°C”). **A**. Levels of chlorophyll *a* and chlorophyllide *a* per gram fresh weight of leaves. **B**. Chlorophyllide *a* levels in sample extracts expressed as the ratio of chlorophyllide *a* to the sum of chlorophyll *a* and chlorophyllide *a.* Chl *a*, chlorophyll *a*. Chlide *a*, chlorophyllide *a*. Error bars indicate standard deviations. Sample size, n = 3.

Schenk et al. [23] have already demonstrated that chlorophyllide formation could be minimized if leaf samples were ground in liquid nitrogen and extracted with acetone cooled to −20°C. In this study, we attempted to simplify their method. We evaluated whether or not chlorophyll could be extracted just by immersing frozen leaves in pure acetone cooled to −30°C. Our data indicated that a substantial amount of chlorophyll remained in leaf tissue after an overnight incubation in −30°C acetone (data not shown). After 4 days of incubation, the majority of chlorophyll had been extracted as evidenced by the white appearance of extracted leaves (data not shown). HPLC analysis showed that only trace amounts of chlorophyllide were detected in samples obtained from WT leaves that had been treated or not with MeJA (Figures 6 and 7), indicating that CLH activity was negligible at −30°C.

### Extraction of chlorophyll by DMF or ethanol

DMF is reported to efficiently extract chlorophyll without the need for homogenization [26]. In the present study, we tested whether or not chlorophyllide formation occurs during extraction with DMF. WT leaves, treated or not with MeJA, were incubated for 12 h in DMF at 4°C. Only a trace amount of chlorophyllide was detected (Figure 7). Modification of pigment structure that impacts the profiles obtained by HPLC separation of major photosynthetic pigments, including chlorophyll *a,* did not occur with the use of DMF (Figure 6).

We also examined the ability of ethanol to extract chlorophyll in this study. WT leaves, those were either treated or not with MeJA, were incubated for 12 h in ethanol at 4°C. This solvent did not extract all of the chlorophyll from leaves after a 12-h incubation, as evidence by the leaves retaining some greenish color. Additionally, this solvent induced significant modifications of the pigments (Figure 6).

### Comparison of the chlorophyll extraction methods in different plant species

**Figure 8:**
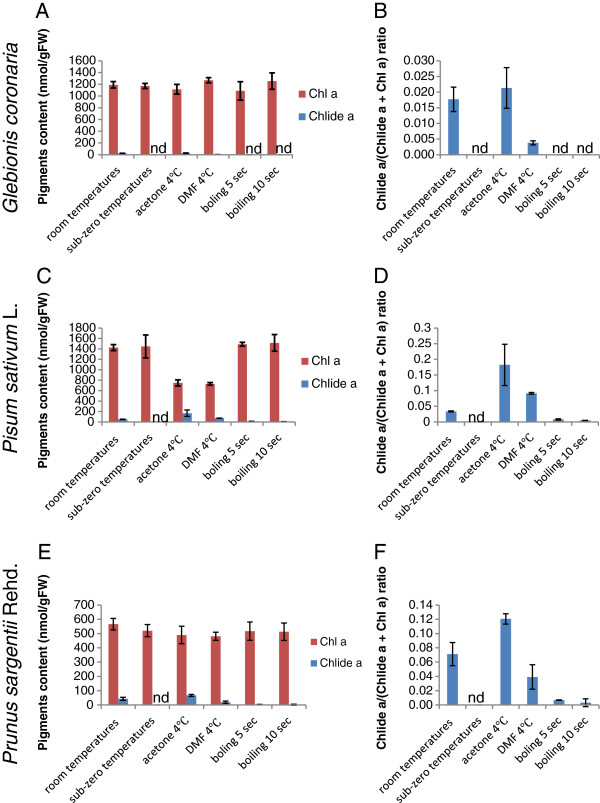
**Comparison of extraction methods for chlorophyllide formation with three different species (*Glebionis coronaria*, *Pisum sativum *L. and *Prunus sargentii *Rehd.).** Leaves were homogenized in acetone at room temperatures (“room temperatures”) or sub-zero temperatures (“sub-zero temperatures”), respectively, by grinding leaves with steel stainless beads. Alternatively, leaves were immersed in pure acetone at 4°C (“acetone 4°C”) or in pure DMF at 4°C (“DMF 4°C”), respectively, without the grinding procedure. In the last pair of experiments, leaves were boiled for 5 (“boiling 5 sec”) or 10 sec (“boiling 10 sec”), and were subsequently immersed in acetone at 4°C for chlorophyll extraction. Details of the methods is described in the Method section. **A**, **C** and **E**. Levels of chlorophyll *a* and chlorophyllide *a* per gram FW of leaves. **B**, **D** and **F**. Chlorophyllide *a* levels in sample extracts expressed as the ratio of chlorophyllide *a* to the sum of chlorophyll *a* and chlorophyllide *a.* Chl *a*, chlorophyll *a*. Chlide *a*, chlorophyllide *a*. Error bars indicate standard deviations. Sample size, n = 3.

For testing the general utility of the three methods as described above, chlorophyll was extracted from the leaves of three other plant species, namely, *Glebionis coronaria* (garland chrysanthemum), *Pisum sativum* L. (pea) and *Prunus sargentii* Rehd. (North Japanese hill cherry) (Figure 8A-F). Chlorophyllide *a* was detected in all three species when pigments were extracted by homogenizing leaves in pure acetone at room temperature or by immersing leaves in 4°C pure acetone. These results indicate that all three species possess CLH activity. Among these species, the largest accumulation of chlorophyllide was observed with pea leaves when the pigments were extracted by immersing leaves in pure acetone at 4°C, which converted 20% of chlorophyll *a* to chlorophyllide *a* (Figure 8D). In contrast, the *G. coronaria* leaves did not show high CLH activity, which yielded only 2% of chlorophyllide *a* compared to total chlorophyll *a* levels by the acetone immersion method (Figure 8B). The sub-zero temperature extraction yielded negligible amounts of chlorophyllide from all three samples (Figure 8A-F), demonstrating that the chlorophyllide formed during the other extraction methods was predominantly an artifact. The boiling method worked well with the leaves of *G. coronaria,* which formed negligible amounts of chlorophyllide *a* (Figure 8A and B). This method resulted in the formation of small amounts chlorophyllide *a* with pea and cherry leaves (Figure 8C-F). The chlorophyllide *a* levels in this method were in a similar range with Arabidopsis, which was approximately 1% or less of total chlorophyll *a* (Figure 8D and F). The DMF extraction method did not work as well for *G. coronaria,* pea and cherry leaves as it did for Arabidopsis. This method allowed the formation of chlorophyllide *a* up to 10% of total chlorophyll *a* in pea leaves (Figure 8D). Moreover, immersing leaves in DMF (48 h) and acetone only extracted half of the pigments in comparison to other methods (Figure 8C). Interestingly, a short boiling before immersing leaves in pure acetone drastically improved the extraction efficiency of pigments (Figure 8C).

## Discussion

It has been reported that the commonly used method of extraction of photosynthetic pigments with aqueous acetone sometimes results in artifactual chlorophyllide formation [18,23]. In the present study, we determined the quantity of chlorophyllide formation before, during or after extraction of these pigments using several different methods of extraction. By suppressing CLH activity during extraction, we demonstrated that only trace amounts of chlorophyllide, if any, are present in cells prior to extraction (see Figures 4, 5, 7 and 8). We also showed that both chlorophyll and chlorophyllide are stable in acetone after extraction (Table 1). Therefore, it is unlikely that chlorophyllide is formed in the solvents after the extraction procedure is completed. Based on our collective results, we concluded that chlorophyllide is formed during the extraction process. We speculate that chlorophyllide is formed when acetone infiltrates the tissue, or when the tissue is homogenized in acetone. During these processes, the actual concentration of acetone to which cells are exposed may increase gradually rather than immediately, thus allowing an opportunity for aberrant enzymatic reactions to occur. Although CLH is known to precipitate in pure acetone, it is capable of remaining highly active in lower concentrations of aqueous acetone [18,27]. Therefore, it is likely that CLH catalyzes the formation of chlorophyllide during extraction until the actual acetone concentration reaches nearly 100%. This hypothesis explains the differential effects of DMF on chlorophyllide formation during chlorophyll extraction from different plant species (Figures 7 and 8). DMF suppressed chlorophyllide formation in Arabidopsis leaves almost perfectly, while it allowed chlorophyllide formation in other plant species whose leaves are thicker than Arabidopsis (Figure 8). These observations can be explained by the assumption that the infiltration of DMF occurs more slowly in thicker leaves as compared to in thinner leaves.

The aforementioned hypothesis raises the question why CLH is only active after the tissue is homogenized with organic solvents or soaked in organic solvents. A possible answer to this question may be that CLH is active in cells but separated from chlorophyll in cells. Schenk et al. [23] used CLH-GFP targeting experiments and confirmed that CLH is localized outside of chloroplasts. If CLH is indeed separated from chlorophyll in intact cells, the homogenization of cells or immersion of tissue in acetone may disrupt cell structures and enable CLH to act on chlorophyll.

Chlorophyllide has been long considered to be an intermediate of both chlorophyll biosynthesis and breakdown [20,28]. Hörtensteiner and co-workers [23,29], however, suggested that chlorophyllide is not a true intermediate of chlorophyll breakdown, at least during leaf senescence in Arabidopsis. Instead, they indicated that chlorophyll is degraded via pheophytin (Figure 1B). Our results are consistent with the chlorophyll breakdown model of Hörtensteiner and coworkers [23,29]. The majority of chlorophyllide detected in acetone extracts of leaf pigments in our experiments were formed by the action of CLH during extraction (see Figures 3 and 4). These results suggest that plants only accumulate a small amount (if any) of chlorophyllide in cells under either normal growth conditions ([23] and this study) or when exposed to MeJA.

We compared three methods that suppress CLH activity with a conventional acetone-extraction method. In the first method, Arabidopsis leaves were boiled for a short time (5 or 10 sec). This procedure almost completely suppressed chlorophyllide formation with Arabidopsis and *G. coronaria* leaves. Bacon and Holden [17] already reported that a 5 minute period of boiling eliminates chlorophyllide formation. Their boiling time, however, appears to have been too long since they observed extensive decomposition of the pigments [17]. In principle, the boiling time used in this procedure should be optimized for each plant species but we do not suggest boiling leaves for more than 10 sec for most plant species (see Figure 8). Thicker leaves may necessitate a longer boiling time. For example, we found that a 30 sec boiling time worked well to eliminate CLH activity in mulberry leaves in our laboratory (data not shown). This method appears to have another advantage in increasing the extraction efficiency of pigments from thicker leaves such as pea leaves when pigments are extracted by immersing leaves in organic solvents (see Figure 8C). Thus, the boiling method combined with the use of DMF as an extractant would be worth testing when pigments are extracted from thicker leaves. A possible drawback of the boiling method is the potential for additional types of modification to chlorophyll molecules. For instance, we observed a slight increase in pheophytin *a* concentration in our extracts (Figure 6) indicating that 0.1 to 0.2% chlorophyll *a* might be converted to pheophytin *a* by boiling. Thus, the boiling method is recommended in studies where the quantitation of pheophytin *a* is not being considered.

In the second method, frozen leaves were ground at sub-zero temperatures in a metal box that was cooled with liquid nitrogen. The leaves were then homogenized in pure acetone cooled to −30°C using an automatic bead shaker, Shake Master. The use of this shaker facilitates the processing of a relatively large number of samples. It is also possible to use cooled mortar and pestles for grinding leaves at sub-zero temperatures. However, this approach may be laborious and time-consuming when the analysis of a large number of samples is required. In addition, the recovery of a sufficient amount of solvent from a mortar can be problematic when only a small amount of sample tissue is used or available [24]. Therefore, the usage of a mortar and pestle with this method is recommended only when a relatively small number of samples need to be analyzed and when a sufficient amount of tissue is available for each sample. Another limitation of this method will be a requirement of liquid nitrogen, which might not be readily available in field research. Regardless of these limitations, this method is superior to other methods in completely suppressing CLH activity in all plant species tested in this study. This method would be suitable for determining the minimum levels of chlorophyllide formation.

In the third method, pigments were extracted with DMF. This solvent has been previously used for pigment extraction [24,26] but, to the best of our knowledge, was not tested for chlorophyllide formation. This solvent prevents CLH activity even during an overnight incubation of Arabidopsis leaves at 4°C (Figure 7). Therefore, the use of DMF appears to be the best option for extracting photosynthetic pigments from this model organism for downstream analysis using HPLC without introducing artifacts. However, this solvent is not as effective for *G. coronaria,* pea and cherry leaves as it is for Arabidopsis leaves (Figure 8). Moreover, this solvent is a possible liver toxin [30] and all appropriate safety guidelines should be adhered to in its use. Although the volatility of DMF is low, it should be carefully handled in an exterior venting fume hood. In conclusion, the use of DMF might be restricted to Arabidopsis or similar plant species under well-ventilated laboratory conditions.

## Conclusions

We demonstrated that the most-widely used acetone-based procedures for the extraction of photosynthetic pigments from leaf samples potentially results in the rapid, artifactual conversion of chlorophyll to chlorophyllide, especially when pigments are extracted from leaves with high amounts of CLH. This alteration affects HPLC analysis of photosynthetic pigments by decreasing the apparent content of chlorophyll in extracts. The artifactual conversion can be prevented or reduced by adopting one of three simple methods described in this study, namely, short-time boiling of samples prior to extraction with acetone, extraction at sub-zero temperatures, and the use of DMF as a solvent. A researcher may consider one of the three extraction methods depending on the plant material, availability of equipment or liquid nitrogen, and the purposes of pigment analysis.

## Methods

### Chemicals

Acetone (HPLC grade, 99.7% purity) was purchased from Wako Pure Chemical Industries, Ltd, Osaka, Japan. DMF (Guaranteed Reagent Grade), ethanol (HPLC grade) and other solvents (Guaranteed Reagent Grade) were purchased from Nacalai Tesque, Inc., Kyoto, Japan.

#### Plant materials

Wild-type Arabidopsis (Columbina-0 ecotype) and a T-DNA insertion line (SALK_124978; designated as *clh1-1,*[23]) were primarily used in this study. Plants were grown in soil under long-day conditions (16 h light/8 h dark) in growth chambers under fluorescent light (70–90 μmol photons m^-2^s^-1^) at 23°C. For pigment extraction, leaves (7th to 9th leaves counting from the bottom of the plant) were harvested either after 4 weeks or after a period of 8 weeks to allow natural senescence. For the MeJA treatment, 4-week-old plants were sprayed with 100 μM MeJA in 0.1% ethanol, 0.01% Tween 20, or solvent control (0.1% ethanol, 0.01% Tween 20), and kept for 3 days at the same growth conditions. For dark-induced senescence, the 7th-9th leaves of 4-week-old plants were detached and placed on wet filter paper (3 mM MES buffer, pH 5.8, with or without 50 μM MeJA) and incubated in complete darkness at 23°C for up to 3 days. In addition to Arabidopsis, three other plant species were tested in this study. *Glebionis coronaria* (garland chrysanthemum) adult plants were purchased from a supermarket, and their mature leaves were used for the experiments. *Pisum sativum* L. (pea) was grown in soil under long-day conditions (16 h light/8 h dark) for seven days in growth chambers under fluorescent light (70–90 μmol photons m^-2^s^-1^) at 23°C. Then, young leaves were harvested for pigment extraction. Young leaves of *Prunus sargentii* Rehd. (North Japanese hill cherry) were collected from the campus of Hokkaido University, Japan in mid-May.

#### Chlorophyll extraction and analysis

Leaves were harvested and the fresh weight (18–30 mg) of each sample was recorded. Leaves were then frozen in liquid nitrogen and stored at −80°C in a deep freezer. In most experiments described in this study, pigments were extracted by immersing leaves in organic solvents for 10 to 48 hours. Incubation time and the organic solvent were varied from experiment to experiment, which is described in the result section. The procedure described below is common to all extraction methods used in this study unless otherwise noted. Firstly, tubes with leaves were removed from the liquid nitrogen and 1 ml of organic solvents cooled to 4°C or −30°C was immediately added to each tube and incubated at 4°C in the dark for 12 h for Arabidopsis, 20 h for *G. coronaria*, 48 h for pea and 10 h for cherry leaves. The time length of incubation was determined for each plant species by preliminary experiments. For Arabidopsis leaves, the results of longer incubation in acetone at −30°C in the dark for 4 days were also described in the results section. The 80% acetone employed in this study contained 20% (v/v) 0.2 M Tris–HCl pH 8.

In the boiling method, the leaves were dipped into boiling water for 5 or 10 sec. The leaves were then placed on filter paper to absorb excess water and then homogenized in pure acetone at room temperature. Alternatively, four-degree acetone was added to the boiled samples in a 2-ml microtube. Tubes were then kept at 4°C for 12 h for Arabidopsis, 20 h for *G. coronaria*, 48 h for pea and 10 h for cherry leaves. After incubation, extracts were transferred to a glass vial and analyzed using HPLC as described below.

In the second extraction method, acetone was cooled to −30°C prior to its use. An aluminum metal box (BioMedical Science Co. Ltd., Tokyo, Japan) for holding sample tubes during shaking was cooled in liquid nitrogen for 30 min prior to its use. Two-ml microcentrifuge tubes containing leaf samples and homogenization beads were frozen in liquid nitrogen and then stored at −80°C Acetone was added to the tubes containing the frozen samples while they were in the nitrogen-cooled metal box. Homogenization of the tissue was performed immediately by shaking the sample tubes containing the homogenization beads and leaf samples in an automatic bead shaker (Shake Master, BioMedical Science Co. Ltd, Tokyo, Japan).

In the third extraction method, 1 ml of DMF cooled to 4°C was immediately added to frozen leaves. The samples were subsequently incubated at 4°C in the dark for 12 h for Arabidopsis, 20 h for *G. coronaria*, 48 h for pea and 10 h for cherry leaves. Subsequently, the organic solvent was recovered by centrifugation and its pigment composition was determined by HPLC as described below.

### HPLC separation of photosynthetic pigments

The microtubes containing the homogenized samples were subjected to centrifugation at 15,000 rpm for 5 min at 4°C and the resulting supernatant was analyzed by HPLC with a Symmetry C8 column (150 mm in length, 4.6 mm in i.d.; Waters, Milford, MA, USA) according to the method of Zapata et al. [31]. Elution profiles were monitored by measuring absorbance at 410 nm. Pigments used as standards were purchased from Juntec Co. Ltd. (Odawara, Japan).

### Time course experiments

Leaf samples from 4-week-old Arabidopsis plants were immersed in pure acetone in 2.0 mL-microtubes that were held in a metal box that had been pre-cooled with liquid nitrogen. The sample tubes were then transferred to a tube rack and incubated at ambient temperature for the times indicated in Figure 4. After a prescribed time, the tubes were returned to the nitrogen-cooled metal box to terminate the incubation. Pigments were extracted from samples at a sub-zero temperature while tubes were in the cooled metal box by adding stainless beads and shaking the box in a bead shaker (Shake Master).

## Abbreviations

CLH: Chlorophyllase; DMF: N, N’-dimethylformamide; FW: Fresh weight; HPLC: High performance liquid chromatography; MeJA: Methyl-jasmonate.

## Competing interests

The authors declare that they have no competing interests.

## Authors’ contributions

The experiments were conceived by XH, AT and RT, and performed by XH. The manuscript was written by XH and RT. RT is the principal investigator of the research grant. All authors read and approved the final manuscript.
